# Effects of bitter almond on production performance, antioxidant capacity and immune function of Rongde black-feathered small-sized layer strain

**DOI:** 10.3389/fvets.2025.1678499

**Published:** 2025-10-10

**Authors:** Jun-Nan Chen, Yi-Fan Chen, Han Guo, Er-Ying Hao, Lei Shi, Hui Chen, Xiang-Yu Chen, Ya-Peng Ma, De-He Wang, Li-Jun Xu

**Affiliations:** ^1^College of Animal Science and Technology, Hebei Agricultural University, Baoding, China; ^2^Baoding City Animal Husbandry Work Station, Baoding, China; ^3^Hongwei Agricultural Technology Co., Ltd., Baoding, China

**Keywords:** bitter almond, hens, metabolomics, egg quality, antioxidant capacity

## Abstract

**Introduction:**

Bitter almond, as a natural plant-derived additive, possesses the potential to enhance antioxidant and immune functions. Furthermore, its rapid metabolism *in vivo* leads to low residual levels. However, its effects on laying hens’ production performance and health remain unclear.

**Methods:**

A total of 180 healthy 43-week-old Rongde black-feathered small-sized layer strain (RBSL) with similar production performance were selected and randomly divided into four groups (with five replicates per group). These groups were fed diets containing 0 (control group, CON), 0.25 (low-dose group, LBA), 0.5 (medium-dose group, MBA), and 0.75 (high-dose group, HBA) g/kg of bitter almond, respectively, for an 8-week experiment period.

**Results:**

The results showed that in terms of production performance. The MBA group exhibited a significantly higher laying rate, daily feed intake, and Haugh unit than the CON group (*p* < 0.05). The HBA group also showed greater Haugh unit and yolk color versus controls (*p* < 0.05). In terms of antioxidant and immune functions, the T-AOC, GSH-Px, and IgA levels in the MBA and HBA groups were significantly higher than those in the CON group (*p* < 0.05), while the IgM level was significantly increased only in the HBA group (*p* < 0.05). In terms of intestinal morphology, LBA, MBA, and HBA groups all significantly improved the intestinal morphology of RBSL, with the MBA group showing the most pronounced improvement (*p* < 0.05). Additionally, metabolomics analysis revealed that bitter almond powder altered plasma metabolite profiles. KEGG enrichment analysis indicated that these alterations affected pathways including ABC transporters and tumor choline metabolism (*p* < 0.05). Meanwhile, microbiome analysis showed that bitter almond powder modified the cecal microbial community structure, notably resulting in a significant decrease in the abundance of the genus *Negativibacillus* in the HBA group. Furthermore, the abundance of *Negativibacillus* was significantly positively correlated with levels of IgA, IgM, and GSH-Px (*p* < 0.05).

**Discussion:**

In conclusion, bitter almond supplementation improves egg production, along with antioxidant and immune status, as well as intestinal microbiota. Considering comprehensive benefits and safety, 0.5 g/kg is the optimal addition dosage, which can improve production performance without showing potential toxicity risks.

## 1 Introduction

Since the 1940s, antibiotics have been added to livestock and poultry feed in Europe and the United States, with up to 80% of antibiotics in the US being used in animal production (1). However, macrolide antibiotics such as tilmicosin exhibit concentrations exceeding 2000 ng/kg in poultry pectoral muscles, livers, and kidneys on the first day, requiring approximately 30 days for complete metabolic clearance (2). This situation has necessitated the search for potential alternatives to antibiotics in feed (China banned their use in feed starting in 2020) (3).

Plant-based feed additives (including alkaloids, terpenoids, and phenolic compounds) have significantly lower residue levels than antibiotics (such as, curcumin can be excreted within 48 h), and they are gaining attention due to their antioxidant and immune-boosting properties (4–6). Bitter almonds, as a plant-based feed additive, primarily contain amygdalin (1.14–5.07%), protein (15.7–24.1%), fatty acids (32.2–43.2%), minerals (2.51–3.83%), and flavonoids (1.2%) as active components (7–9). Bitter almonds and their extract, amygdalin, have been widely applied in the livestock industry. Sahil and colleagues found that amygdalin can enhance the total antioxidant capacity, free radical scavenging ability, and globulin levels of broiler hens, thereby improving their antioxidant and immune capabilities (10); Kim et al., demonstrated that incorporating varying levels of almonds significantly improved production performance in broiler chickens, including feed conversion ratio and nutrient digestibility (11); Mahboub et al., demonstrated that different concentrations of bitter almond can enhance antioxidant and immune markers such as immunoglobulin M, total antioxidant capacity, and glutathione peroxidase in carp (12); Chen et al., found that amygdalin can alleviate oxidative stress and inflammation by inhibiting the transforming growth factor-β1/Smad signaling pathway in rats (13); Ma et al., also found that amygdalin alleviates airway damage by inhibiting abnormal changes in airway epithelial structure and apoptosis (14). Additionally, studies have shown that 10,000 μg/mL bitter almond glycoside can regulate ovarian hormone production in pigs by stimulating the release of estradiol-17 beta (15). In summary, bitter almonds, as a plant-derived additive, hold potential for enhancing antioxidant and immune functions. Due to their rapid metabolism and low residue levels (16), they align with the green development trend of antibiotic alternatives in animal husbandry, making them a natural additive with significant development potential.

However, amygdalin also has adverse effects. For example, it can hydrolyze into cyanide in the small intestine, which may cause tissue hypoxia at high doses and subsequently lead to metabolic acidosis (17). Additionally, it remains unclear whether supplementing diets with bitter almond can improve laying hen performance, antioxidant capacity, and immune function. Therefore, this study is the first to evaluate the effects of incorporating varying doses of bitter almonds into laying hen diets on growth performance, egg quality, antioxidant capacity, and immune function. The study will help determine the optimal dosage for use as a feed additive and provide a theoretical foundation for developing natural plant-based feed additives. At the same time, we predict that adding bitter almond powder to feed can significantly enhance the antioxidant and immune functions of laying hens, thereby improving their production performance.

## 2 Materials and methods

### 2.1 Experimental materials

The bitter almond (amygdalin content: 3.59%) was provided by Yongshengtang Pharmaceutical Co., Ltd. (Bozhou, Anhui, China). The amygdalin content was determined by Hebei Agricultural University using high-performance liquid chromatography (Agilent 6495C, Agilent Technologies, Inc., Santa Clara, CA, United States). Rongde black-feathered small-sized layer strain (RBSL) (a breed approved in 2023) has a daily feed intake of 90–100 g/day at 40 weeks of age, with a feed-to-egg ratio of 2.05–2.10:1.

### 2.2 Experimental design

The animal use protocols in this experiment were approved by the Ethics Committee on Experimental Animals of Hebei Agricultural University (approval no.: 20241115; Baoding, Hebei, China), in compliance with the principles of animal protection, welfare, and ethics.

The feeding experiment was conducted in environmentally controlled chambers at the Animal Husbandry Teaching Base of Hebei Agricultural University. A total of 180 healthy 43-week-old Rongde black-feathered small-sized layer strain (RBSL) with similar production performance and body weight were selected and randomly divided into 4 groups (each group with 5 replicates, each containing 9 hens). All experimental diets in this study were isonitrogenous and isocaloric. They were, respectively, fed basal diets supplemented with 0 (control group, CON), 0.25 (low-dose group, LBA), 0.5 (medium-dose group, MBA), and 0.75 (high-dose group, HBA) g/kg of bitter almond. Bitter almond was added in powder form to the basal diet and ensured to be homogeneously mixed. The experiment included a 3-day observation period, a 1-week adaptation period, and an 8-week formal experimental period. The hens were housed in H-type stepped cages with ad libitum access to feed and water. They received complete formulated diets twice daily (08:00 and 16:00) under a 16-h light: 8-h dark (16 L:8D) lighting regimen. Daily at 15:30, the number of eggs laid, flock condition, mortality, and culling rate were recorded. The composition and nutritional levels of the basal diet are shown in Table 1.

**Table 1:** Composition and nutritional levels of the basal diet (%, air-dried base).

Diet composition	Content (%)	Nutritional levels^2^	Content (%)
Corn	59.30	ME/(MJ/kg)	11.62
Soybean meal	24.00	CP	16.64
Limestone	7.00	Ca	3.45
Soybean oil	1.00	TP	0.39
Premix^1^	5.00	Lys	0.90
Fish meal	0.20	Trp	0.24
Wheat bran	3.50	Thr	0.77
		Ile	0.89
Total	100.00	Met	0.37

(1) Nutrient content provided per kilogram of premix.

(2) Reference standard for nutrient content.

### 2.3 Sample collection

#### 2.3.1 Production performance

Daily records were kept of the egg production number and egg weight for each replicate. Weekly measurements were taken of the feed intake for each group. Using the above data, the egg production rate, average egg weight, feed-to-egg ratio, and average daily feed intake were calculated. The calculation formulas are as follows: laying rate (%) = (total number of eggs produced/total number of hens) × 100; average egg weight (g) = total weight of eggs/number of eggs; average daily feed intake (g) = total feed consumption/number of experimental days; and feed-to-egg ratio (F/G) = total feed consumption/total egg weight.

#### 2.3.2 Egg quality

At 2, 4, 6, and 8 weeks of age, 6 eggs were randomly selected from each group (a total of 30 eggs per group) for testing. First, egg weight and short axis/long axis were recorded. Eggshell strength was measured using an eggshell strength tester (ESTG-01; Israel Aoke Company Limited, Israel), and Haugh units and yolk color were determined using an automatic egg quality analyzer (EA-01; Israel Aoke Company Limited, Israel). Eggshell thickness was measured using a digital spiral micrometer (217–111, Guilin Guanglu Digital Measurement and Control Co., Ltd. Guilin, Guangxi, China) at the blunt end, sharp end, and midpoint, and the average value was calculated. Egg yolk weight and eggshell weight were obtained by weighing with an electronic balance.

#### 2.3.3 Serum antioxidant and immune indices

On day 7 of week 8 of the experiment at 8:00 a.m., hens were weighed after an 8-h fast. Ten hens were randomly selected from each group. Blood was collected from the wing vein, placed in a coagulation tube, and left at room temperature for 4 h. The serum was then separated by centrifugation at 3000 revolutions per minute for 15 min. An enzyme-linked immunosorbent assay kit (Nanjing Jiancheng Biotechnology Research Institute, Nanjing, Jiangsu Province, China) was used to detect serum levels of the following parameters: antioxidant markers, including glutathione peroxidase (GSH-Px), superoxide dismutase (SOD), malondialdehyde (MDA), total antioxidant capacity (T-AOC), and catalase (CAT); and immunoglobulins, including immunoglobulin A (IgA), immunoglobulin M (IgM), and immunoglobulin G (IgG). This method is based on the immunological principle of antigen–antibody specific binding, utilizing enzyme-labeled technology to amplify detection signals. During the experiment, capture antibodies coated onto a solid-phase carrier first bind to the target antigen. Subsequently, enzyme-labeled detection antibodies are added to form a complex. Finally, through enzyme-catalyzed substrate colorimetric reactions, quantitative analysis is achieved by leveraging the property that absorbance values are directly proportional to target concentration.

#### 2.3.4 Intestinal morphology analysis

At 16:00 on day 7 of week 8, 2 hens were randomly selected from each replicate (totaling 40 hens) for slaughter. The duodenum, jejunum, and ileum were isolated, and the intestinal contents were removed. The tissues were then rinsed with physiological saline. Approximately 2 cm mid-segment samples were collected from each intestinal region and fixed in 4% paraformaldehyde for 48 h. Subsequently, the samples were embedded in paraffin and sectioned for histological analysis. All subsequent steps, from sectioning onward, including staining and morphological analysis, were performed under blinded conditions. Hematoxylin–eosin (H&E) staining was performed, and the sections were examined under a digital trinocular camera microscope (BA210Digital, Motic China Group Co., Ltd., Xiamen, Fujian, China). villus height and crypt depth were measured for each intestinal segment, and the villus height-to-crypt depth ratio (villus height/crypt depth ratio) was calculated. The measurement method for intestinal histology was performed according to Kiernan (18).

#### 2.3.5 Plasma untargeted metabolomics

On day 7 of week 8, randomly select 4 plasma samples from each group, take 1 milliliter, lyophilize it, and then add 100 microliters of an 80% methanol aqueous solution. The mixture was vortexed and incubated on ice for 5 min. Centrifugation was performed at 15,000 rpm and 4° C for 15 min. The supernatant was diluted with mass spectrometry-grade water to a methanol concentration of 53%, and the mixture was centrifuged again under the same conditions for 15 min. The supernatant was collected and analyzed by liquid chromatography-mass spectrometry (LC–MS). Chromatography was performed using a Hypersil Gold column (C18) at 40° C with a flow rate of 0.2 mL/min. The mobile phase in positive ion mode consisted of 0.1% formic acid (phase A) and methanol (phase B), while in negative ion mode, it consisted of 5 mM ammonium acetate (pH 9.0, phase A) and methanol (phase B). The raw data were converted to mzXML format using ProteoWizard, followed by peak matching, retention time correction, and peak area extraction using XCMS software (Scripps Research, La Jolla, CA, United States). Subsequently, metabolite structural identification, data preprocessing, experimental data quality evaluation, and data analysis were performed sequentially, with false discovery rate (FDR) correction applied for multiple comparisons during statistical testing.

#### 2.3.6 Microbial community structure of cecal contents

On day 7 of week 8, four cecal content samples were randomly selected from each group. Approximately 150–200 mg of each sample was transferred to a 2 mL centrifuge tube, and 1.2 mL of Buffer SSL was added. Vortex the mixture for 1 min and then incubate at 70° C for 10 min. After vortexing again for 15 s, centrifuge the samples at 14,000 rpm for 10 min. From the resulting supernatant, 250 μL was aliquoted, then add 20 μL of Proteinase K and 250 μL of Buffer AL. Invert the mixture to mix and incubate at 70° C for 10 min. Then, add 250 μL of anhydrous ethanol and mix thoroughly. Transfer the mixture to the Hi Pure DNA Mini Column I, wash with Buffer GW1 and Buffer GW2, and centrifuge. Subsequently, add 50–200 μL of preheated Buffer AE, incubate at room temperature for 2 min, and centrifuge to collect the DNA. The purity of the DNA was assessed using Nano Drop, with acceptable values defined as an A260/A280 ratio between 1.8–2.0 and an A260/A230 ratio ≥ 2.2, while integrity was verified by 2% agarose gel electrophoresis. The V3–V4 region of the 16S rDNA was amplified with V341F/V806R primers in a 50 μL reaction system under the following conditions: 95° C for 5 min; 30 cycles of 95° C for 1 min, 60° C for 1 min, and 72° C for 1 min; followed by a final extension at 72° C for 7 min. After detection, purification, and quantification, the amplified products were used for library preparation with an Illumina kit, and library quality was evaluated using ABI StepOnePlus real-time PCR. The raw reads obtained from sequencing were initially processed using the FASTP software for quality control to remove low-quality reads. Subsequently, paired-end sequencing reads were assembled into tags using the FLASH software. The assembled tags then underwent further filtering to generate high-quality data, designated as clean tags. Using these clean tags, OTU clustering was performed at a 97% similarity threshold with the UPARSE algorithm implemented in the USEARCH software. During the clustering and alignment process, chimeric sequences were detected and removed using the UCHIME algorithm within USEARCH, resulting in effective tags for subsequent analysis. Following OTU generation, the effective tags were utilized for OTU abundance statistics, followed by taxonomic annotation, *α*-diversity analysis, *β*-diversity analysis, and other downstream.

### 2.4 Statistical analysis

Laying rate, egg quality, antioxidant indicators, immune indicators, and intestinal morphology data were first organized using Microsoft Excel 2020, then subjected to one-way analysis of variance using Statistical Package for the Social Sciences software. For indicators where the ANOVA revealed a significant overall effect, differences between individual groups were compared using Duncan’s new multiple range test. Results are presented as “mean ± standard deviation” (*p* value less than 0.05 indicates significant difference); graphs were created using GraphPad Prism 8.0 (GraphPad Software Incorporated, San Diego, CA, United States), with *p* value less than 0.05 indicating significant difference. Additionally, plasma metabolomics and gut microbiota data were analyzed using nonparametric tests (Kruskal-Wallis test) in combination with multivariate statistical methods. Spearman correlation analysis was used to examine the interrelationships among plasma metabolomics, intestinal microbiota, immunoglobulins, and antioxidant parameters.

## 3 Results

### 3.1 Production performance

**Figure 1:**
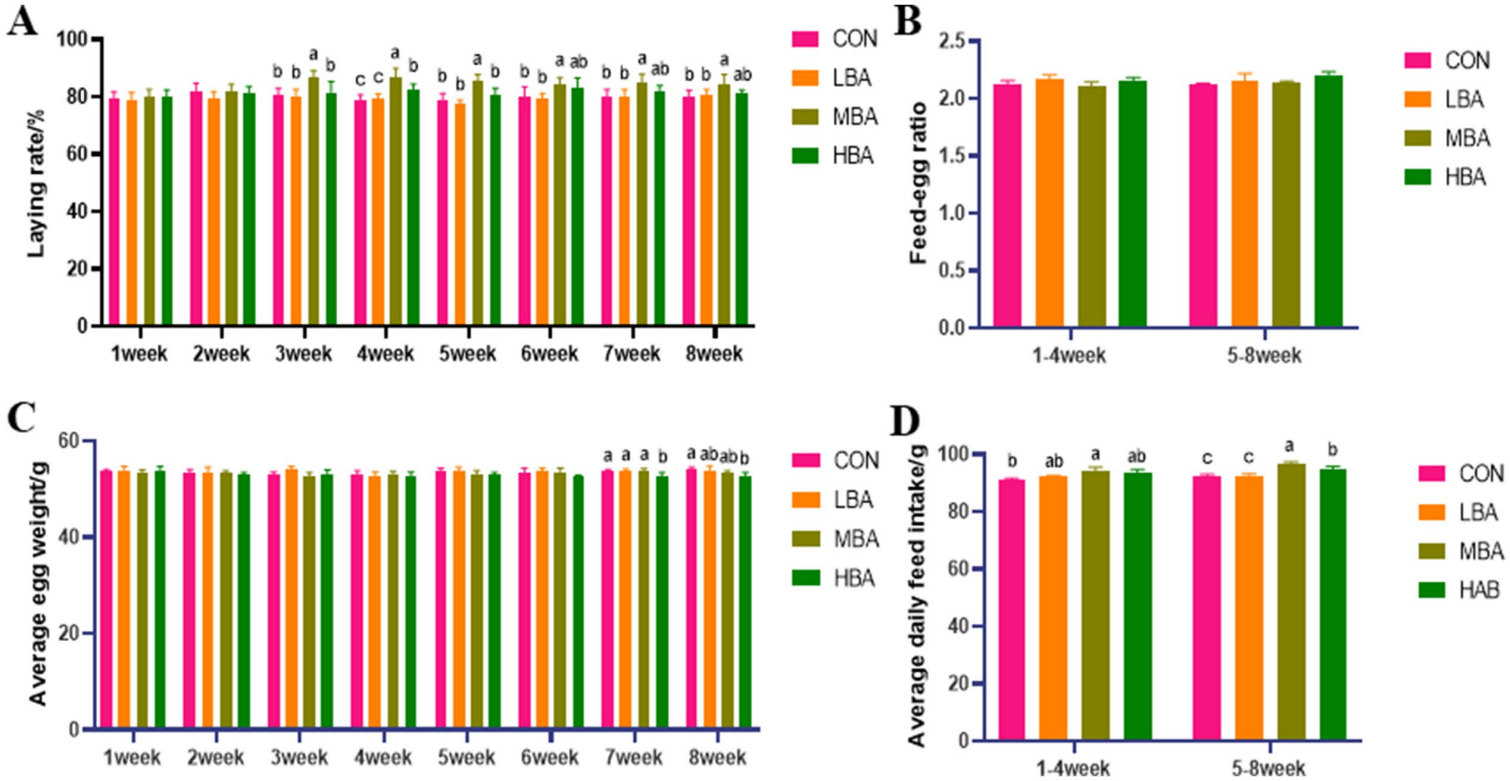
Effects of bitter almond on the production performance of RBSL. **(A)** Laying rate in control group (CON), low-dose bitter almond group (LBA), medium-dose bitter almond group (MBA), and high-dose bitter almond group (HBA); **(B)** feed-egg ratio; **(C)** average egg weight; **(D)** average daily feed intake. Results are presented such that no superscript letters or identical superscript letters indicate no significant difference (*p* > 0.05), while different lowercase superscript letters indicate significant differences (*p* < 0.05).

As shown in Figure 1A, the egg production rates in the MBA group were significantly higher than those in the CON group from weeks 3 to 8 (*p* < 0.05). In contrast, the average egg weight in the HBA group was significantly lower than that in the CON group during weeks 7 to 8 (*p* < 0.05; Figure 1C). Additionally, the MBA group exhibited a significantly higher average feed intake than the CON group throughout the entire experiment (*p* < 0.05; Figure 1D). Meanwhile, no significant differences were observed in the feed-to-egg ratio among the four groups (*p* > 0.05; Figure 1B).

### 3.2 Egg quality

**Figure 2:**
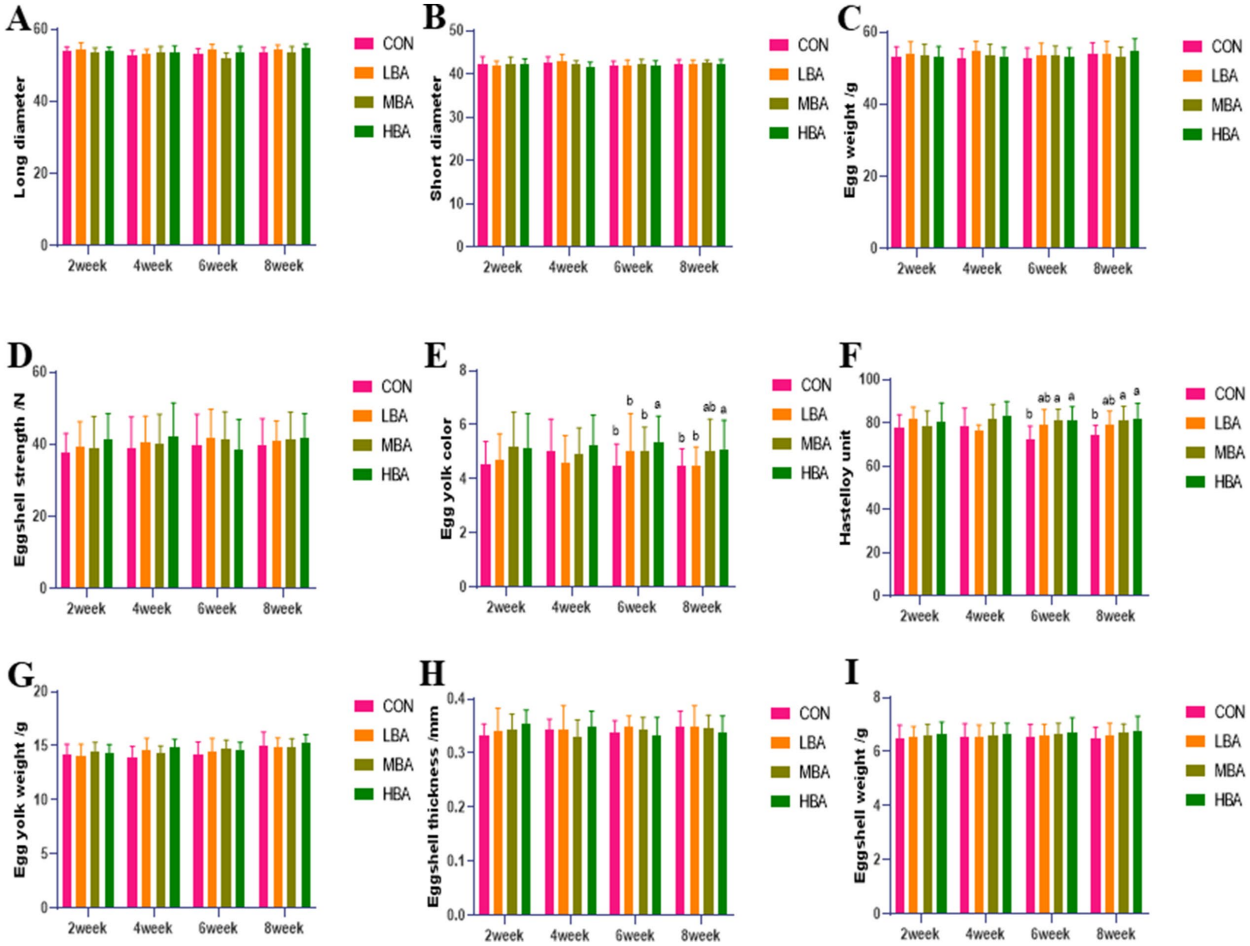
Effects of bitter almond on the egg quality of RBSL. **(A)** Long axis **(B)** short axis **(C)** egg weight **(D)** eggshell strength **(E)** yolk color **(F)** Haugh unit **(G)** yolk weight **(H)** eggshell thickness **(I)** eggshell weight. Among these, CON represents the control group, LBA represents the low-dose bitter almond group, MBA represents the medium-dose bitter almond group, and HBA represents the high-dose bitter almond group. The results indicated that the absence of letters or identical letters signifies no significant difference (*p* > 0.05), while different lowercase letters indicate significant differences (*p* < 0.05).

As shown in Figures 2E,F, during weeks 6 to 8, the yolk color of the HBA group was significantly higher than that of the CON group (*p* < 0.05). The Haugh units of both the MBA and HBA group were significantly higher than those of the CON group (*p* < 0.05). No other egg quality indicators differed significantly among the four groups (*p* >  0.05; Figures 2A–D,G–I).

### 3.3 Analysis of serum antioxidant capacity and immunoglobulin levels

**Figure 3:**
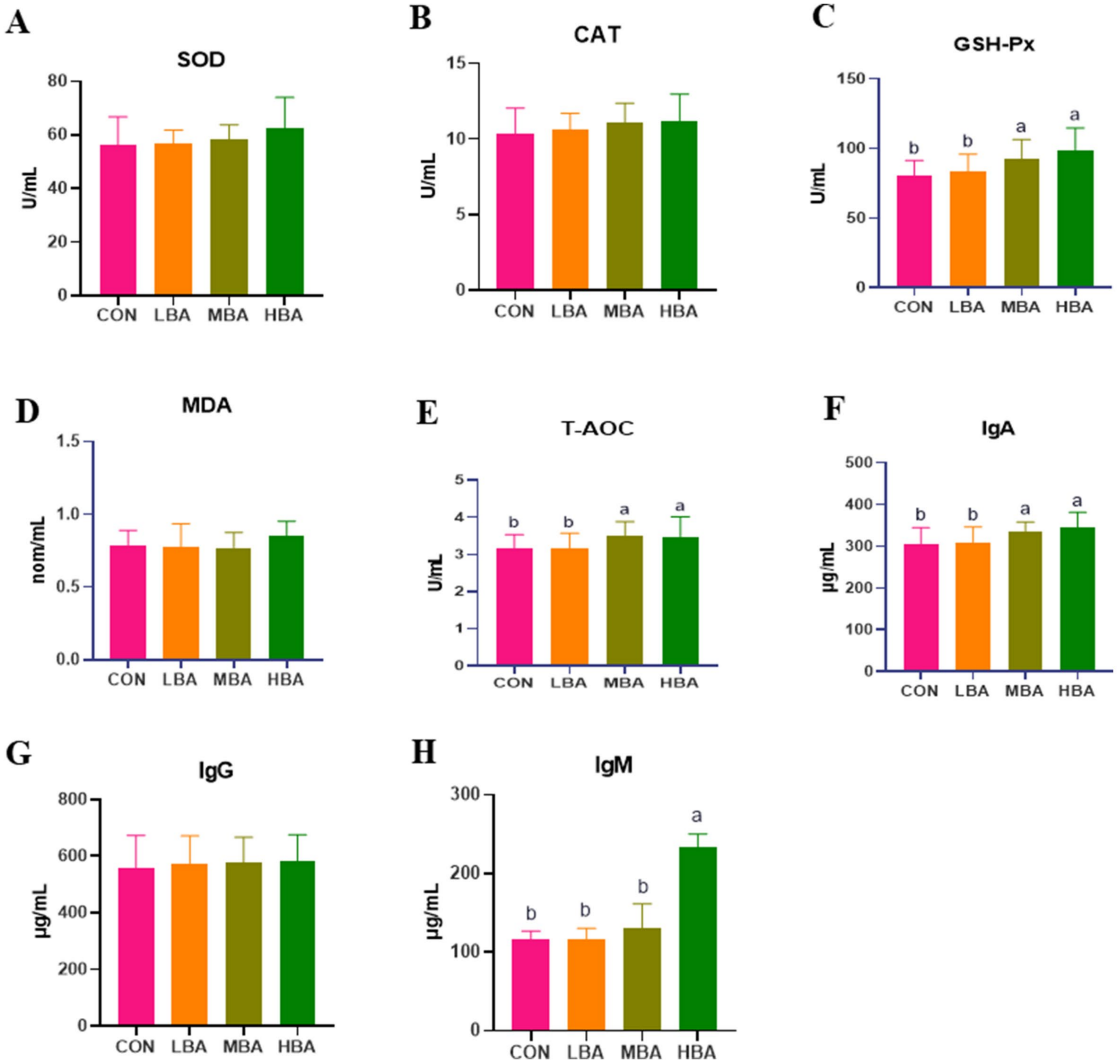
Effects of bitter almond on serum antioxidant capacity and immune function in RBSL. **(A)** Superoxide dismutase (SOD) activity; **(B)** catalase (CAT) activity; **(C)** glutathione peroxidase (GSH-Px) activity; **(D)** malondialdehyde (MDA) content; **(E)** total antioxidant capacity (T-AOC); **(F)** immunoglobulin A (IgA) content; **(G)** immunoglobulin G (IgG) content; **(H)** immunoglobulin M (IgM) content. Among these, CON represents the control group, LBA represents the low-dose bitter almond group, MBA represents the medium-dose bitter almond group, and HBA represents the high-dose bitter almond group. The results indicated that the absence of letters or identical letters signifies no significant difference (*p* > 0.05), while different lowercase letters indicate significant differences (*p* < 0.05).

Figure 3 illustrates the effects of bitter almond powder on antioxidant and immune parameters in RBSL. The levels of T-AOC, GSH-Px, and IgA in the MBA and HBA groups were significantly higher than those in the CON group (*p* < 0.05; Figures 3C,E,F). IgM levels were significantly elevated only in the HBA group (*p* < 0.05), showing a dose-dependent increase (Figure 3H). However, no significant differences were observed in SOD, CAT, MDA, or IgG levels among the four groups (*p* > 0.05; Figures 3A,B,D,G).

### 3.4 Intestinal morphology

**Figure 4:**
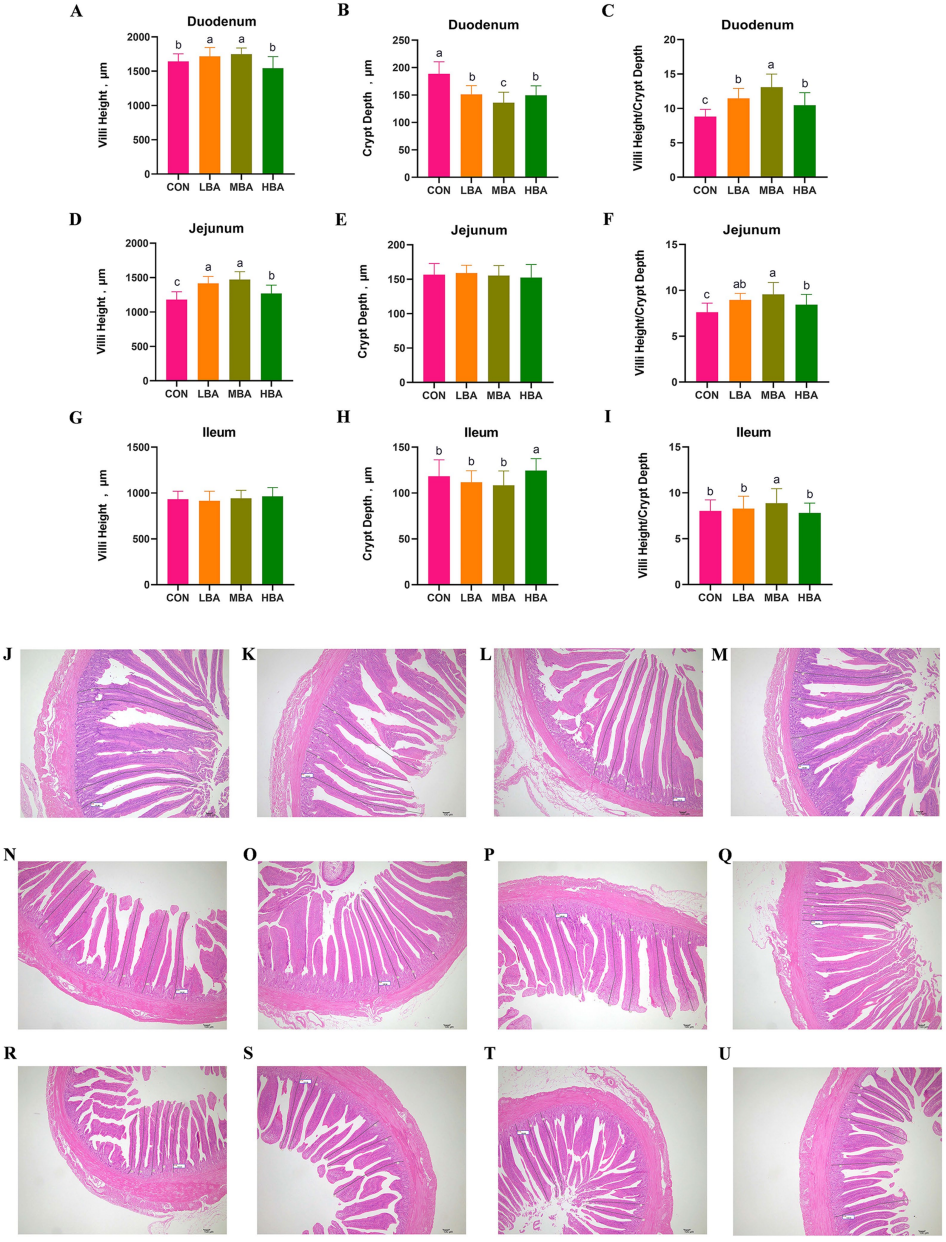
Effects of bitter almond on the intestinal morphology of RBSL. **(A)** Duodenal villus height (VH), **(B)** duodenal crypt depth (CD), **(C)** ratio of duodenal villus height to crypt depth (V/C), **(D)** jejunum villus height (VH), **(E)** jejunum crypt depth (CD), **(F)** ratio of jejunum villus height to crypt depth (V/C), **(G)** ileum villus height (VH), **(H)** ileum crypt depth (CD), and **(I)** ratio of ileum villus height to crypt depth (V/C). Among these, CON represents the control group, LBA represents the low-dose bitter almond group, MBA represents the medium-dose bitter almond group, and HBA represents the high-dose bitter almond group. The results indicated that the absence of letters or identical letters signifies no significant difference (*p* > 0.05), while different lowercase letters indicate significant differences (*p* < 0.05). **(J)** Representative duodenal images from the CON group; **(K)** representative duodenal images from the LBA group; **(L)** representative duodenal images from the MBA group; **(M)** representative duodenal images from the MBA group. **(N)** Representative images of the jejunum in the CON group; **(O)** representative images of the jejunum in the LBA group; **(P)** representative images of the jejunum in the MBA group; **(Q)** representative images of the jejunum in the HBA group. **(R)** Representative images of ileum from the CON group; **(S)** representative images of ileum from the LBA group; **(T)** representative images of ileum from the MBA group; **(U)** representative images of ileum from the HBA group.

As shown in Figures 4A,D, the villus height in the duodenum and jejunum was significantly greater in the LBA and MBA groups than in the CON group (*p* < 0.05). Meanwhile, the crypt depth in the duodenum of the LBA, MBA, and HBA groups was significantly lower than that of the CON group (*p* < 0.05; Figure 4B). Moreover, the villus-to-crypt ratio in the duodenum and jejunum of these three groups was significantly higher than that of the CON group (*p* < 0.05; Figures 4C,F). In the ileum, crypt depth was significantly greater in the HBA group than in the CON group, while the villus-to-crypt ratio was significantly higher in the MBA group than in the control group (*p* < 0.05; Figures 4H,I). There were no significant differences in the crypt depth of the jejunum or the villus height of the ileum among the four groups (*p* > 0.05; Figures 4E,G). Figures 4J–M shows representative duodenal images for CON, LBA, MBA, and HBA, respectively; Figures 4N–Q shows representative jejunal images for CON, LBA, MBA, and HBA, respectively; Figures 4R–U shows representative ileal images for CON, LBA, MBA, and HBA, respectively.

### 3.5 Plasma untargeted metabolomics analysis

**Figure 5:**
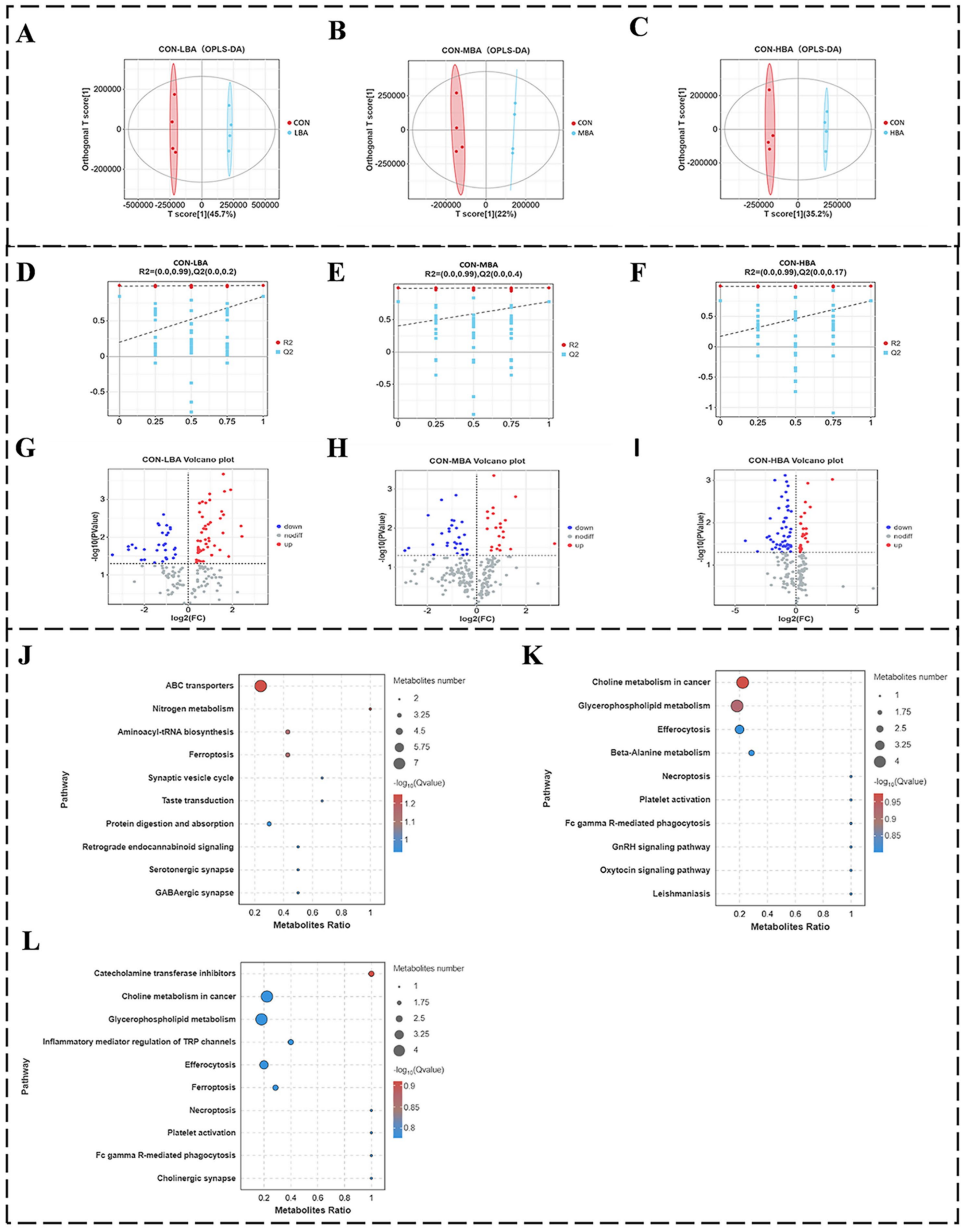
Effects of bitter almond on the cecal bacterial community structure of RBSL. **(A–C)** shows OPLS-DA score plots; **(D–F)** displays permutation test plots for pairwise comparisons; **(G–I)** presents volcano plots of differential metabolites; **(J–L)** illustrates KEGG significance bubble plots. Among these, CON represents the control group, LBA represents the low-dose bitter almond group, MBA represents the medium-dose bitter almond group, and HBA represents the high-dose bitter almond group.

Figure 5 illustrates the differences in plasma metabolites among the four groups through non-targeted metabolomics analysis. Figures 5A–C show the results of Pairwise comparison OPLS-DA analysis, indicating that samples from the CON group are significantly separated from those in the LBA, MBA, and HBA groups along the first principal component (PC1) axis, with all samples falling within the 95% confidence ellipse. The PC1 contribution rates for Figures 5A–C are 45.7, 22, and 35.2%, respectively. Additionally, the results of pairwise comparison permutation tests for each group in Figures 5D–F show that the R^2^Y intercept values for Figures 5D–F are all 0.99, while the Q^2^Y values are 0.2, 0.4, and 0.17, respectively. Since the R^2^ values for all groups are greater than the Q^2^ values, the models are suitable for subsequent analysis. Subsequently, differential metabolite screening was performed based on three parameters: VIP, FC, and *p*-value. The thresholds were set as VIP > 1.0, *p*-value < 0.05, and Fold Change (FC) ≥ 1. As shown in Figures 5G–I, volcano plot analysis of differentially expressed metabolites identified 81, 49, and 78 differentially expressed metabolites in the CON vs. LBA, CON vs. MBA, and CON vs. HBA comparisons, respectively. Among these, the numbers of upregulated metabolites were 51, 21, and 26, while the numbers of downregulated metabolites were 30, 28, and 52, respectively. As shown in Figures 5J–L, further metabolic pathway enrichment analysis revealed that the LBA group significantly affected ABC transporters, nitrogen metabolism, and aminoacyl-tRNA biosynthesis (*p* < 0.05). The MBA group exhibited alterations in pathways such as choline metabolism in cancer, glycerophospholipid metabolism, and Efferocytosis (*p* < 0.05). The HBA group showed changes in pathways including catecholamine transferase inhibitors, choline metabolism in cancer, and glycerophospholipid metabolism (*p* < 0.05).

### 3.6 Bacterial community structure of cecal contents

**Figure 6:**
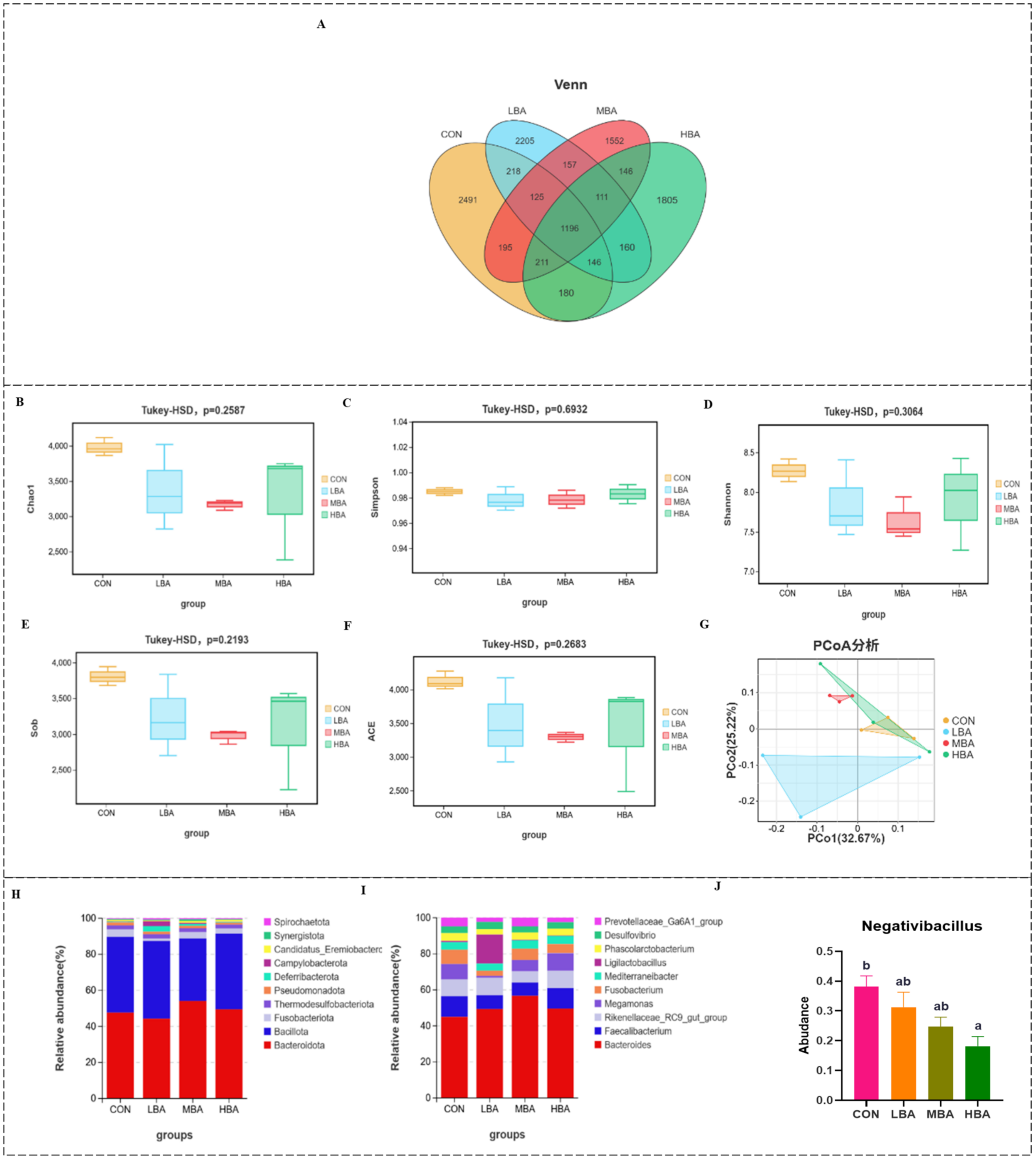
Effects of bitter almond on the bacterial community structure in the cecal contents of RBSL. **(A)** Venn diagram of OTUs in the cecal microbiota; **(B–F)** *α*-diversity of the cecal microbiota (Chao1, Simpson, Shannon, Sob, ACE); **(G)** *β*-diversity of the cecal microbiota; **(H)** species stacking plot of the cecal microbiota at the phylum level; **(I)** species stacking plot of the cecal microbiota at the genus level; **(J)** influence of the cecal microbiota (genus level). Among these, CON represents the control group, LBA represents the low-dose bitter almond group, MBA represents the medium-dose bitter almond group, and HBA represents the high-dose bitter almond group.

To investigate the effects of bitter almond on the microbial community of the cecum in RBSL, microbial changes were measured in the CON, LBA, MBA, and HBA groups. The numbers of OTUs specific to the CON, LBA, MBA, and HBA groups were 2,491, 2,205, 1,552, and 1,805, respectively (Figure 6A). *α*-Diversity was measured using the Chao1, Simpson, Shannon, Sob, and ACE indices. As shown in Figures 6B–F, there were no significant differences in the α-diversity of the cecal microbiota among the CON, LBA, MBA, and HBA groups (*p* > 0.05). Figure 6G shows that there was a certain degree of dispersion among the four groups in the principal component analysis. Figures 6H,I illustrate the effect of bitter almond on the relative abundance of cecal microbiota. At the phylum level, the main phyla were Bacteroidota, Bacillota, Fusobacteriota, Thermodesulfobacteriota, and Pseudomonadota. At the genus level, the main genera were *Bacteroides*, *Faecalibacterium*, *Rikenellaceae_RC9_gut_group*, *Megamonas* and *Fusobacterium*. Among these, the relative abundance of the genus *Negativibacillus* was significantly lower in the HBA group compared to the CON group (*p* < 0.05), and the LBA and MBA groups also showed a decreasing trend (Figure 6J).

**Figure 7:**
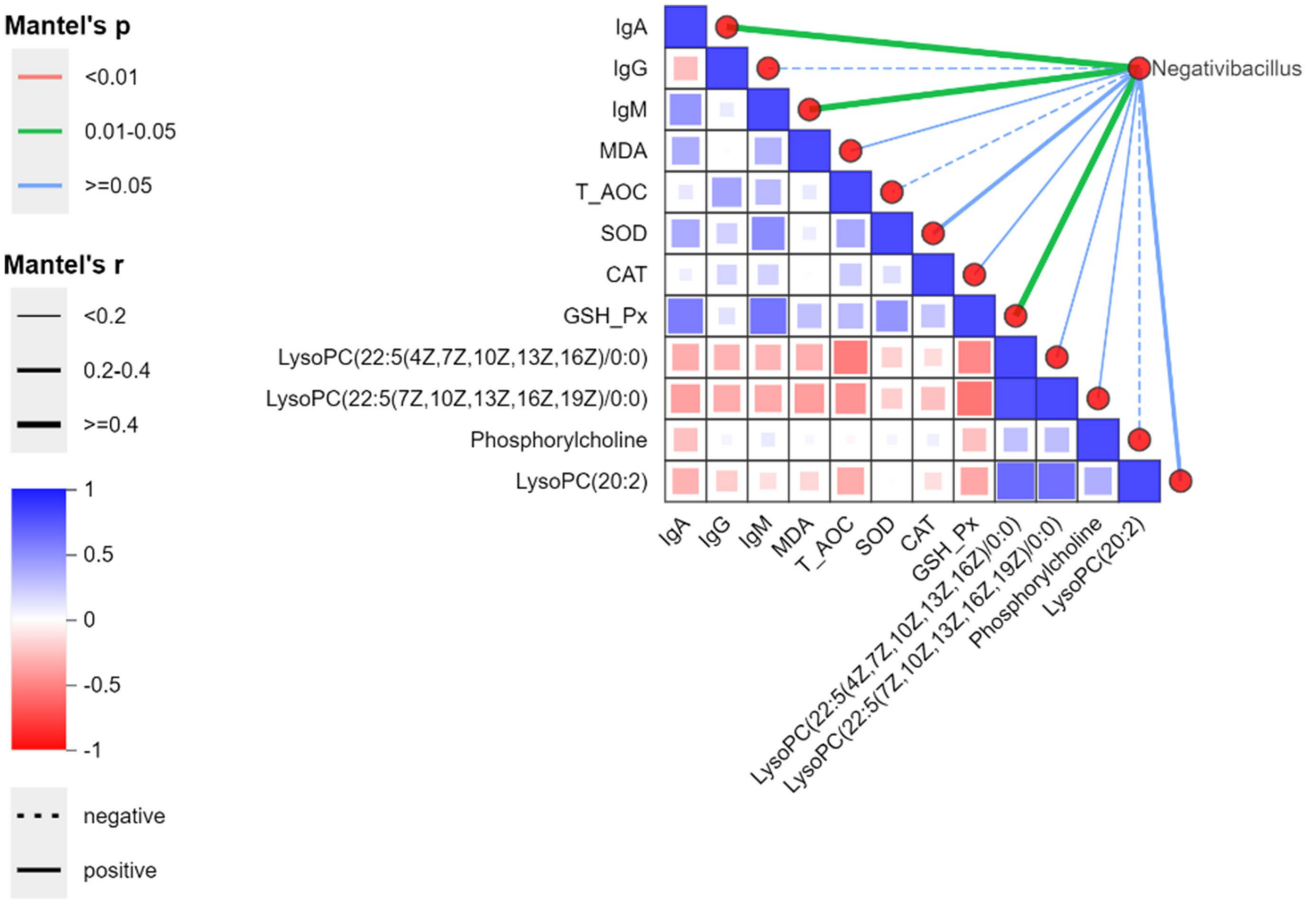
Spearman correlation heatmap at the genus level between bitter almond and RBSL. The color gradient represents Spearman correlation coefficients (red for positive correlations, blue for negative correlations). On the right, the network diagram displays correlations between environmental factors and species based on the Mantel test, where line width indicates the absolute value of the correlation coefficient (Mantel’s r), color represents the signifpicance *p*-value range of (Mantel’s p), and line style (solid/dashed) distinguishes between positive and negative correlations.

Based on antioxidant and immune results, we selected differential genera from the Bacillota phylum (*Negativibacillus*), differential metabolites in the tumor choline metabolism pathway (a metabolic pathway significantly enriched in both the MBA and HBA groups), antioxidant indicators, and immune indicators, and used Spearman test to analyze overall correlations. As shown in Figure 7, the genus *Negativibacillus* was significantly positively correlated with IgA, IgM, and GSH-Px. This is related to the fact that residual antigens still maintain immune stimulation after their abundance decreases.

## 4 Discussion

### 4.1 Effects of bitter almond on the production performance of RBSL

Production performance is a key indicator for evaluating the economic efficiency of laying hens, directly reflecting the production potential and economic benefits of the flock (19). The study found that replacing part of the animal fat with 4% full-fat bitter almonds in broiler chicken diets significantly improved feed conversion ratio and nutrient digestibility (11). Additionally, adding 1% cherry kernels (containing amygdalin) to the diet can improve broiler chickens’ average feed intake, carcass traits, and meat quality (20). The results of this experiment are similar to those of previous studies, indicating that the MBA and HBA groups exhibited a significant increase in average feed intake. Furthermore, this study found that the MBA group significantly increased the laying rate of RBSL, which may be attributed to the antioxidant and immune-enhancing effects of amygdalin promoting follicle development (21). Studies have shown that adding 200–300 mg/kg of magnolol (a compound with antioxidant properties) (22) or 20 mg/kg of vitamin E (VE) (23) can significantly increase laying rates in hens.

Notably, the average egg weight in the HBA group showed a significant decrease, a phenomenon potentially linked to the accumulation of hydrogen cyanide (HCN) resulting from the metabolism of excessive amygdalin within the body. Existing toxicological research indicates that hydrogen cyanide specifically inhibits the activity of cytochrome c oxidase in cellular mitochondria, thereby causing respiratory suppression in tissue cells and disrupting oxidative phosphorylation. This cellular-level energy metabolism disorder leads to severe insufficiency in adenosine triphosphate (ATP) synthesis, resulting in functional hypoxia or even apoptosis in cells (24). During egg production, this subacute state of cell apoptosis may further impair the efficiency of yolk precursor protein synthesis in the liver and reduce the nutrient deposition capacity of the oviduct (25), which may ultimately lead to a significant decrease in egg weight.

### 4.2 Effects of bitter almond on the egg quality of RBSL

Haugh units are an important indicator for assessing the viscosity and freshness of egg whites. The higher the Haugh unit value, the better the viscosity of the egg white, egg yolk color is an indicator of yolk pigmentation (26) and is generally associated with the carotenoid content in the feed (27). Carotenoids are inherently prone to oxidation, which can disrupt protein molecular structures (e.g., peptide bond cleavage and cross-linking) and lead to egg white thinning (28). Studies have shown that adding 1 and 4% black chokeberry to feed can reduce pigment oxidation loss in laying hens and improve yolk color (29). Additionally, adding 10 g/kg of green tea meal can inhibit free radical-induced oxidative damage to egg white proteins, maintain their natural structure, and thereby enhance egg yolk color and Haugh units (30). The results of this experiment are similar to those found by previous researchers, indicating that the egg yolk color and Haugh units in the MBA and HBA groups were significantly higher than those in the CON group.

### 4.3 Effects of bitter almond on serum antioxidant capacity and immune function in RBSL

Under normal conditions, the level of free radicals in poultry is maintained at a relatively stable level by the body’s antioxidant system, which plays an important physiological role in normal life activities (31). Recent studies have confirmed that amygdalin in bitter almond can enhance the body’s antioxidant capacity by reducing the overproduction of free radicals caused by infection (32). Studies have shown that supplementing rats with 3 g/kg of amygdalin can alleviate endoplasmic reticulum stress induced by tunicamycin and exhibit certain antioxidant activity (33). The results of this study demonstrated that, compared with the CON group, the MBA and HBA groups showed significantly increased GSH-Px and T-AOC levels, and SOD and CAT activity exhibited a dose-dependent upward trend. Immunoglobulins, as important immune effector molecules in animal humoral immunity, directly reflect the regulatory effect of feed nutrition on immune function (34). Research indicates that the immunomodulatory effects of amygdalin are primarily mediated through enhanced macrophage phagocytic function and promotion of immune cell proliferation (35). Other studies have shown that amygdalin (25–400 μg/mL) can promote the secretion of IL-2 and interferon (36). The results of this study showed that, compared with the CON group, the MBA group had significantly increased IgA levels, and the HBA group showed further significant increases in both IgA and IgM levels, similar with the results of previous studies. In conclusion, adding bitter almond to feed can enhance the antioxidant and immune capacity of hens to a certain extent.

### 4.4 Effects of bitter almond on the intestinal morphology of RBSL

Intestinal morphological characteristics are important indicators for assessing digestive and absorptive functions, with villus height, crypt depth, and their ratio directly influencing nutrient digestion and absorption efficiency (37, 38). The results of this study demonstrate that the LBA, MBA, and HBA groups improved intestinal morphology in laying hens to varying degrees. Previous studies have shown that different doses of amygdalin (12.5, 25, and 50 mg/kg) can significantly improve intestinal morphology (39). Some indicators in the HBA group showed non-significant or partially decreasing trends, which may be attributed to amygdalin exceeding the detoxification metabolic capacity mediated by intestinal bacteria (40).

### 4.5 Effects of bitter almond on the cecal bacterial community structure of RBSL

This study demonstrates that amygdalin significantly enhances the body’s antioxidant capacity and immune function by regulating key metabolic pathways. Metabolomics analysis revealed that, compared with the CON group, the ABC transporter pathway in the LBA group exhibited significant changes (*p* < 0.05), involving seven metabolites, such as D-chiro-inositol, betaine, and choline. Among them, the supplementation of D-chiral inositol (150 mg/kg) has been shown to alleviate oxidative stress (41). Choline and betaine exhibit synergistic effects in enhancing production performance; specifically, substituting choline with betaine increases egg production in hens by 3.5% (42). The addition of 0.5–1.0% L-glutamic acid and L-glutamine improves both intestinal and immune function (43). Phenylalanine can inhibit inflammatory damage to the lungs (44). The MBA and HBA groups significantly influenced the tumor choline metabolism pathways (*p* < 0.05), promoting the accumulation of four metabolites, including LysoPC (20:2) and LysoPC (22:5(7Z, 10Z, 13Z, 16Z, 19Z)/0:0). Both LysoPC (20:2) and LysoPC (22:5(7Z, 10Z, 13Z, 16Z, 19Z)/0:0) belong to the LysoPC family. Regulating the levels of LysoPC in the blood spectrum can enhance the body’s antioxidant and immune capacities (45). Additionally, LysoPC (22:5) ethanolamine (200 μg/kg) can alleviate mitochondrial damage (46). Furthermore, phosphatidylcholine in the pathway, as a precursor of lecithin, elucidates the mechanism by which adding 2% lecithin improves poultry production performance through its metabolic changes (47). These results collectively indicate that amygdalin dynamically regulates key effector molecules such as D-chiro-inositol, betaine, and LysoPC through ABC transporters and choline metabolism in cancer, thereby activating the antioxidant system, enhancing immune function, and improving production performance. This provides potential insights into the dose–response mechanism of amygdalin at the metabolic level.

### 4.6 Effects of bitter almond on the bacterial community structure in the cecal contents of RBSL

This study assessed the *α*-diversity of the cecal microbiota using the Ace, Chao, Sob, Shannon, and Simpson indices. The results indicated that the addition of bitter almond had no significant effect on α-diversity and richness, but the LBA and MBA groups showed a downward trend. This may be related to the detoxification metabolism of amygdalin mediated by enteric bacteria. Previous studies have shown that the gut microbiota serves as the first line of defense against amygdalin—induced toxicity (48). Notably, α-diversity in the HBA group recovered compared to the MBA group, suggesting that high doses may have adverse effects. At the phylum level, although there was no significant change in the relative abundance of Bacteroidota, the MBA group showed an upward trend, while the LBA and HBA groups exhibited a decline. This aligns with previous studies that reported a correlation between Bacteroidota and broiler growth performance, suggesting that these changes might be dose-dependent on the additive (40). In this study, there were no significant differences in the relative abundance of the Bacteroidetes phylum among the groups; however, the MBA groups showed an upward trend, while the LBA and HBA group showed a decrease, indicating that these changes may be regulated by the additive dose. At the genus level, Bacteroides abundance showed an increasing trend in the LBA and MBA groups, while *Negativibacillus* abundance was significantly reduced in the HBA group (*p* < 0.05) and exhibited decreasing trends in the LBA and MBA groups. Studies have shown that Bacteroides at a concentration of 1 × 10^11^ CFU/mL can significantly improve egg production and other production performance in laying hens (49). However, *Negativibacillus* can induce inflammation and organ failure, etc. (50). Correlation analysis revealed that the *Negativibacillus* was associated with plasma differential metabolites and antioxidant and immune markers, particularly showing a significant positive correlation with IgA, IgM, and GSH-Px (*p* < 0.05). This may be related to the fact that residual antigens still maintain immune stimulation after their abundance decreases. This result aligns with the microbiota-immune co-regulation mechanism and is consistent with the findings of Zhao et al. (51).

When interpreting the results of this study, the following limitations should be considered: First, the experiment employed only a single local breed over an eight-week intervention period; conclusions should be further validated before extrapolating to other commercial breeds. Second, the limited sample size for microbiome analysis (*n* = 4 per group) may reduce statistical power, particularly for detecting low-abundance species or subtle changes. Finally, safety assessments relied on indirect indicators such as production performance and tissue morphology. Direct measurement of cyanide and its metabolites in blood or tissues was absent, preventing precise quantification of metabolic conversion and toxicokinetic characteristics.

## 5 Conclusion

The addition of 0.5 g/kg bitter almond to the diet significantly increased the laying rate, average daily feed intake, and egg quality of Rongde black-feathered small-sized layer strain (*p* < 0.05), while also enhancing plasma GSH-Px and T-AOC activity, as well as IgA and IgM levels, thereby improving antioxidant capacity and immune function. This additive dosage enhanced production performance without demonstrating the toxic effects associated with amygdalin. Under the experimental conditions, the optimal addition rate of bitter almond in the feed was 0.5 g/kg.

## Data Availability

The original contributions presented in the study are included in the article/supplementary material, further inquiries can be directed to the corresponding authors.
